# Pelvic Inflammatory Disease (PID)

**DOI:** 10.1155/S1064744996000130

**Published:** 1996

**Authors:** David E. Soper

**Affiliations:** Medical University of South Carolina 171 Ashley Avenue Charleston SC 29401-2233 USA


					Infectious Diseases in Obstetrics and Gynecology 4:62 (1996)
(C) 1996 Wiley-Liss, Inc.
Images in Infectious Diseases in Obstetrics and Gynecology
Section Editor: David E. Soper, M.D.
Pelvic Inflammatory Disease
(PID)
David E. Soper, M.D.
Medical University of South Carolina
Charleston, SC 29401
LEGEND TO IMAGES
PID is manifested by a continuum of inflammation from
the lower genital tract to the upper genital tract. Evidence
of this inflammation can be confirmed by performing a
saline preparation of the vaginal secretions to document
a marked increase in the number of inflammatory cells
(A). Evidence ofmucopus further documents lower geni-
tal tract inflammation (B). Laparoscopy visually confirms
the presence of edematous, erythematous fallopian tubes
and a sticky purulent exudate documenting upper genital
tract inflammation (C). Further ascending inflammation
can result in perihepatic adhesions (Fitzhugh-Curtis syn-
drome) (D). (C) 1996 Wiley-Liss, Inc.
!1
D
Images in Infectious Diseases in Obstetrics and Gynecology presents clinically important visual images that a practitioner in
women's health might encounter. If you have a high-quality color or black-and-white photograph or slide representing such
an image that you would like considered for publication, send it with a descriptive legend to David E. Soper, M.D., Medical
University of South Carolina, 171 Ashley Avenue, Charleston, SC 29401-2233.
Images is made possible through an educational grant from Pfizer, Inc.
Received June 19, 1996
Images Accepted June 19, 1996

				

